# Concentrations of Fecal Bile Acids in Participants with Functional Gut Disorders and Healthy Controls

**DOI:** 10.3390/metabo11090612

**Published:** 2021-09-09

**Authors:** Shanalee C. James, Karl Fraser, Wayne Young, Phoebe E. Heenan, Richard B. Gearry, Jacqueline I. Keenan, Nicholas J. Talley, Susan A. Joyce, Warren C. McNabb, Nicole C. Roy

**Affiliations:** 1 The Riddet Institute, Massey University, Palmerston North 4474, New Zealand; shanalee.james@agresearch.co.nz (S.C.J.); karl.fraser@agresearch.co.nz (K.F.); wayne.young@agresearch.co.nz (W.Y.); w.mcnabb@massey.ac.nz (W.C.M.); 2 School of Food and Advanced Technology, Massey University, Palmerston North 4472, New Zealand; 3 AgResearch, Tennent Drive, Palmerston North 4472, New Zealand; 4 High-Value Nutrition National Science Challenge, Auckland 1023, New Zealand; phoebe.heenan@postgrad.otago.ac.nz (P.E.H.); richard.gearry@cdhb.health.nz (R.B.G.); 5 Department of Medicine, University of Otago, Christchurch 8011, New Zealand; 6 Department of Surgery, University of Otago, Christchurch 8011, New Zealand; jacqui.keenan@otago.ac.nz; 7 School of Medicine and Public Health, The University of Newcastle, Callaghan, Newcastle 2308, Australia; nicholas.talley@newcastle.edu.au; 8 School of Biochemistry and Cell Biology and APC Microbiome Ireland, University of College Cork, T12 K8AF Cork, Ireland; s.joyce@ucc.ie; 9 Department of Human Nutrition, University of Otago, Dunedin 9016, New Zealand

**Keywords:** bile acids, irritable bowel syndrome, functional gut disorder, liquid chromatography-mass spectrometry

## Abstract

Bile acids are metabolites involved in nutrient absorption and signaling with levels influenced by dietary intake, metabolic processes, and the gut microbiome. We aimed to quantify 23 bile acids in fecal samples to ascertain if concentrations differed between healthy participants and those with functional gut disorders. Fecal bile acids were measured using liquid chromatography-mass spectrometry (LC-MS) in the COMFORT (The Christchurch IBS cohort to investigate mechanisms for gut relief and improved transit) cohort of 250 participants with Rome IV IBS (IBS-constipation (C), IBS-diarrhea (D), IBS-mixed (M)), functional gut disorders (functional constipation (FC), functional diarrhea (FD)) and healthy controls (FC *n* = 35, FD *n* = 13, IBS-C *n* = 24, IBS-D *n* = 52, IBS-M *n* = 29, and control *n* = 97). Dietary information was recorded to ascertain three-day dietary intake before fecal samples were collected. Fecal bile acid concentrations, predominantly primary bile acids, were significantly different between all functional gut disorder participants and healthy controls (CDCA *p* = 0.011, CA *p* = 0.003) and between constipation (FC + IBS-C) and diarrhea (FD + IBS-D) groups (CDCA *p* = 0.001, CA *p =* 0.0002). Comparison of bile acids between all functional groups showed four metabolites were significantly different, although analysis of combined groups (FC + IBS-C vs. FD + IBS-D) showed that 10 metabolites were significantly different. The bile acid profiles of FD individuals were similar to those with IBS-D, and likewise, those with FC were similar to IBS-C. Individuals with a diarrhea phenotype (FD + IBS-D) had higher concentrations of bile acids compared to those with constipation (FC + IBS-C). Bile acid metabolites distinguish between individuals with functional gut disorders and healthy controls but are similar in constipation (or diarrhea) whether classified as IBS or not.

## 1. Introduction

Bile acids are chemical detergents aiding in the digestion and absorption of nutrients [1] and have a regulatory role in the circulatory system impacting lipid, glucose, nutrient, and energy homeostasis [1]. They also mediate interactions between the host and microbiome via cellular receptors (e.g., farnesoid X receptor (FXR), G-coupled protein receptors, vitamin D receptor) [2,3].

Bile acids are synthesized in hepatocytes from cholesterol via the classic and alternative pathways, producing the primary bile acids cholic (CA) and chenodeoxycholic acid (CDCA) [2]. Primary bile acids are conjugated to either glycine or taurine and stored in the gallbladder [2,4]. Bile acids are excreted from the gallbladder into the small intestinal lumen with the bile flow and are unconjugated in the colon by microbial bile salt hydrolase enzymes [5], then modified to secondary bile acids, deoxycholic acid and lithocholic acid (LCA), by microbial dehydroxylase and dehydrogenase enzymes [2,6]. Some bile acids can be toxic to host and microbiota in excess quantities, and hence the regulation of their concentration and metabolism within the hepatic portal system is tightly controlled [1]. Most bile acids are recycled via the enterohepatic circulation multiple times a day; however, approximately 5% of bile acids escape this process and are further modified bacterially before excretion through feces [1].

The microbial conversion of primary to secondary bile acids can be a multi-step rate-limiting process. Only microbial species possessing the bile salt hydrolase enzyme (for example, some members of the *Bacteroides*, *Clostridium*, *Lactobacillus* and *Bifidobacterium* genera) can deconjugate primary bile acids [5,7]. Disturbances to the gut microbiota composition can affect bile acid deconjugation and modification [5]. Furthermore, the gut microbiome converts secondary bile acids to bacterially modified or ‘tertiary’ bile acids [7].

Thus, the interaction between bile acids, the gut microbiome, and host metabolism is an important homeostatic metabolic process [6]. There is increasing evidence that alterations to bile acid metabolism may be associated with clinical disease, including functional gastrointestinal disorders such as IBS. Bile acid malabsorption (BAM) was associated with IBS-diarrhea and is characterized by increased colonic transit and bowel movements, mucus production, and greater epithelial permeability [8,9,10]. Studies showed increased concentrations of specific primary and secondary fecal bile acids in the plasma and feces of individuals with IBS-D compared to IBS-constipation (IBS-C) and healthy controls [10,11,12,13], however these studies are often limited to either a smaller cohort size that does not incorporate multiple functional gut disorders or a smaller bile acid panel. Recently, it was shown that a major subgroup with IBS-D have BAM, and that those in the severe BAM group had a gut microbial shift that correlated with changes in the fecal metabolome and diet [14]. Another study demonstrated that 25% of IBS-D individuals had increased fecal bile acid concentrations [15] compared to healthy controls. Additionally, those with high fecal bile acids had increased relative abundance of *Clostridia*, and elevated expression of the 7α-hydroxylase (hdhA) gene, the primary enzyme converting cholesterol into bile acids [15]. 

We sought here to report a comprehensive panel of bile acid metabolites across the functional gut disorder spectrum (IBS-C, IBS-D, IBS-M, FC, and FD). We hypothesized that fecal bile acid concentrations will differ between IBS subtypes, functional groups, and healthy controls reflecting a perturbation in the metabolic processing of bile acids in functional gut disorders. Within IBS subtypes, bile acids will have a higher fecal concentration in individuals presenting with diarrhea (IBS-D + FD) rather than constipation (IBS-C + FC) based on the available research showing a link between diarrhea and fecal bile acids. To test these hypotheses, this analysis aimed to quantify 23 bile acids that are implicated in multiple different conversion steps and available as chemical standards in fecal samples collected from a cohort of individuals with functional gut disorders (FC, FD, IBS-C, IBS-D, IBS-M) and healthy controls. 

## 2. Results

A total of 259 fecal samples were analyzed; however, there was incomplete metadata for 9 participants, leaving 250 participants in the final analyses. Two participants were taking cholesterol-lowering medication. Symptom questionnaires based on the Rome Criteria IV classified these 250 participants as FC *n* = 35, FD *n* = 13, IBS-C *n* = 24, IBS-D *n* = 52, IBS-M *n* = 29, and healthy control *n* = 97. 

Table 1 outlines bile acid abbreviations and full, common names. Of the 23 bile acid metabolites measured THDCA, βMCA, TβMCA, TαMCA, GDDCA, UDCA, and TUDCA were below the limit of detection. Therefore 16 bile acids were quantified and included in the further analyses.

Table 2 shows the sex and age of the participants in the COMFORT cohort. A gender effect (*p* = 0.000103) was observed between the subtypes as there was a larger proportion of females in all groups compared to males. Age (*p* = 0.128) did not significantly differ between the groups. The average fecal dry weight percentage for all groups is presented in Table 2. Average fecal dry weight was significantly different within the cohort (*p* = 0.013), where FC and IBS-C had a higher dry weight (%) compared to FD and IBS-D. The dry weight (%) of fecal samples from controls was between that of constipation (FC and IBS-C) and diarrhea groups (FD and IBS-D) while IBS-M was higher than all other groups. Pairwise comparison showed no significant difference between the FC and IBS-C groups or the FD and IBS-D groups (Table 2). 

**Figure 1:**
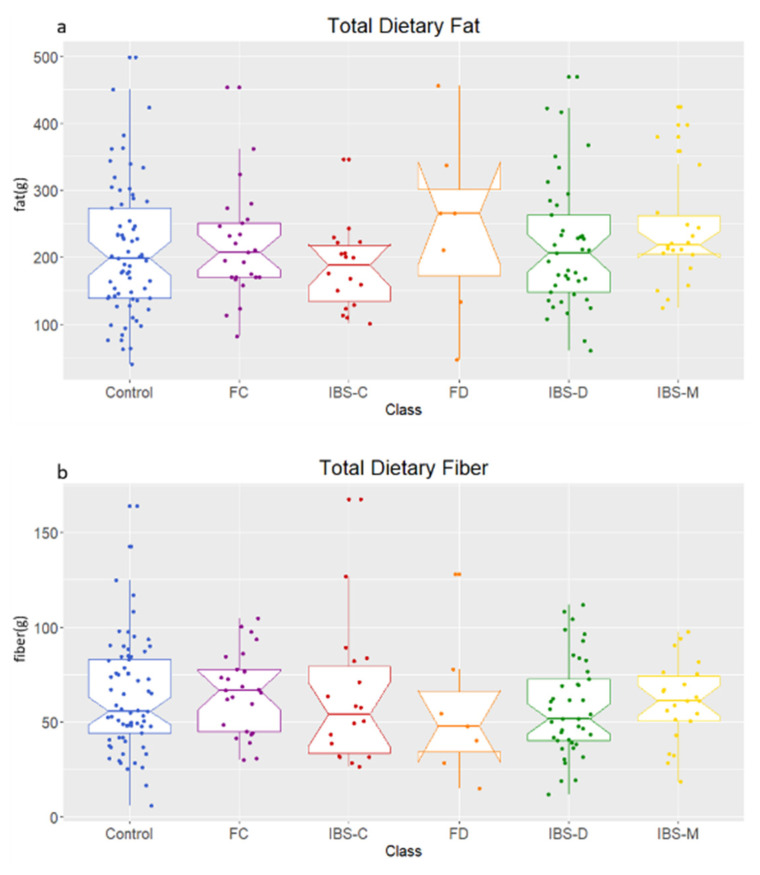
Dietary intake of (**a**) fat and (**b**) fiber over 3-day period recorded using diet diaries for each participant. Healthy control (control), functional constipation (FC), IBS-constipation (IBS-C), functional diarrhea (FD), IBS-diarrhea (IBS-D), IBS-mixed (IBS-M). Boxplots show median (center line), 25th and 75th percentile (top and bottom of boxes, respectively), with whiskers representing 1.5 times the inter-quartile range, and boundaries of notches show 95% confidence interval (CI).

Analysis of 3-day dietary information showed no significant difference in reported fiber (*p* = 0.848) or fat (*p* = 0.401) intake by the participants of the cohort (Figure 1). 

### 2.1. Comparison of Fecal Bile Acid Concentrations between Healthy Control, IBS Subtypes, FC, and FD Groups

**Figure 2:**
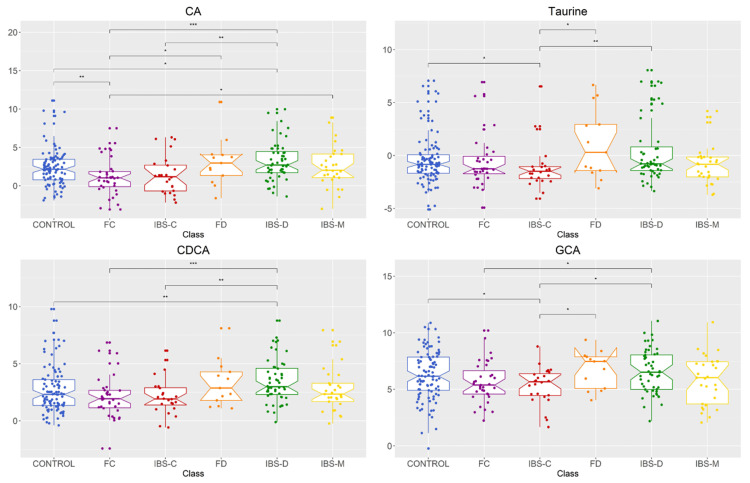
Bile acid metabolite distributions among healthy control, IBS subtypes, and functional gut disorders groups for metabolites with significantly different abundances between groups. Data presented as logged values of µg/mg of fecal dried weight. Healthy control (control), functional constipation (FC), IBS-constipation (IBS-C), functional diarrhea (FD), IBS-diarrhea (IBS-D), IBS-mixed (IBS-M). Boxplots show median (center line), 25th and 75th percentile (top and bottom of boxes, respectively), with whiskers representing 1.5 times the inter-quartile range, and boundaries of notches show 95% confidence interval (CI). Statistical significance denoted as *p* < 0.05 (*), *p* < 0.01 (**), *p* < 0.001 (***). Abbreviations: cholic acid (CA), chenodeoxycholic acid (CDCA), glycol-cholic acid (GCA).

Univariate analysis showed that four bile acid metabolites (CDCA *p* = 0.011, CA *p* = 0.003, GCA *p* = 0.048, taurine *p* = 0.038) were significantly different in fecal concentration between groups (Figure 2, Table A1). Data of all the 16 available bile acids analyzed are shown in Table A2. As shown in Figure 2, further pairwise comparisons were performed for significant metabolites and showed no significant difference between FC and IBS-C groups or FD and IBS-D groups. However, there were significant differences between IBS-C and IBS-D in the fecal concentration of all four metabolites. The differences between healthy control and FC groups were only significantly different for CA. IBS-D and healthy control groups were significant for CA and CDCA. IBS-C and healthy control groups were significant between GCA and taurine. The concentrations of the primary bile acid CDCA was similar between constipation (IBS-C + FC) and diarrhea (IBS-D + FD) groups, respectively (Table A2). Two metabolites were significantly higher in males than females (CDCA *p* = 0.009, HDCA *p* = 0.030).

**Figure 3:**
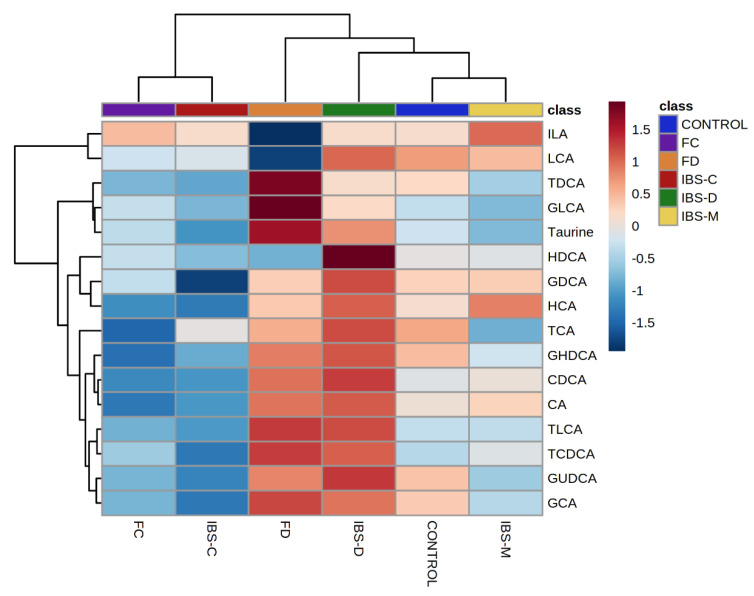
Hierarchical clustering analysis for average values of groups. Healthy control (control), functional constipation (FC), IBS-constipation (IBS-C), functional diarrhea (FD), IBS-diarrhea (IBS-D), IBS-mixed (IBS-M). Data presented as z score of logged values of µg/mg. Color ribbon beneath upper dendrogram identifies groups; healthy control—blue, IBS-C—red, IBS-D—green, IBS-M—yellow, FC—purple, FD—orange.

Hierarchical clustering analysis for group averages showed IBS-D and FD, IBS-C and FC, and healthy controls and IBS-M clustered together (Figure 3). In addition, FD and IBS-D groups clustered separately from the other two groups. FD and IBS-D participants had increased fecal concentrations of bile acids, whilst FC and IBS-C had decreased concentration compared to both IBS-M and healthy controls which were characterized by variable concentrations of bile acids. 

### 2.2. Bile Acid Concentrations Compared between Healthy Control and Combined Functional Groups

As shown in Table 2, Figure 2 and Figure 3, FC and IBS-C and FD and IBS-D, respectively, have similar fecal bile acid profiles and fecal dry weight percentage. Therefore, the datasets from the FC and IBS-C groups were merged into a combined constipation group. Similarly, the datasets of the FD and IBS-D groups were grouped as a combined diarrhea group. Both groups were used to determine if concentration differences in fecal bile acid metabolites could be discerned between healthy controls and those exhibiting constipation or diarrhea symptoms. Additionally, there is uncertainty around the symptoms being experienced by IBS-M participants at the time of fecal sample collection. Thus, further analyses were performed without the IBS-M group. 

**Figure 4:**
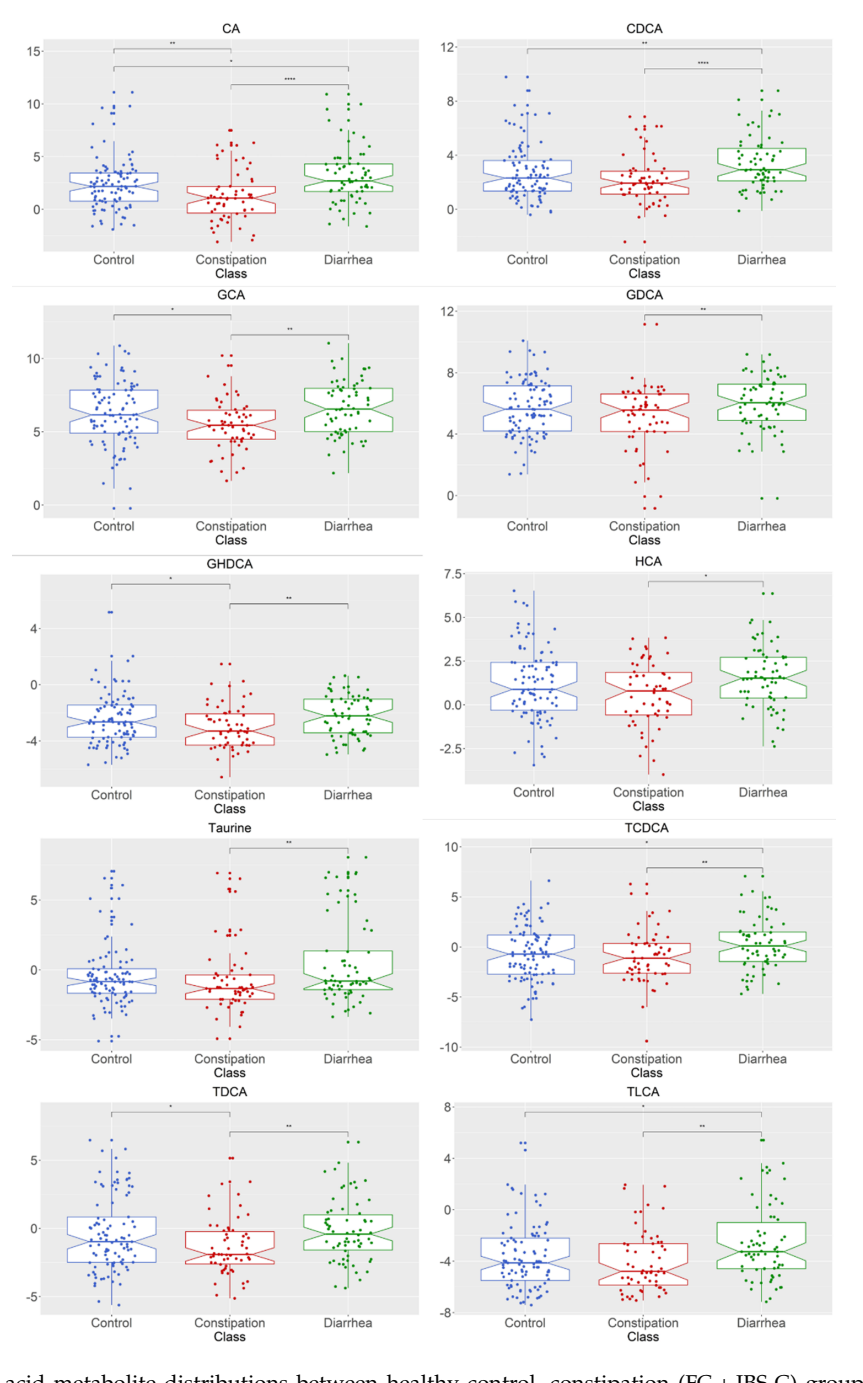
Bile acid metabolite distributions between healthy control, constipation (FC + IBS-C) group, and diarrhea (FD + IBS-D) group for metabolites with significantly different abundances between groups. Data presented as logged values of µg/mg. Boxplots show median (center line), 25th and 75th percentile (top and bottom of boxes, respectively), with whiskers representing 1.5 times the inter-quartile range, and boundaries of notches show 95% confidence interval (CI). Statistical significance denoted as *p* < 0.05 (*), *p* < 0.01 (**), *p <* 0.0001 (****). Abbreviations: cholic acid (CA); chenodeoxycholic acid (CDCA); glyco-cholic acid (GCA); glyco-deoxycholic acid (GDCA); glyco-hyo-deoxycholic acid (GHDCA); hyo-cholic acid (HCA); tauro-cheno-deoxycholic acid (TCDCA); tauro-deoxycholic acid (TDCA); tauro-lithocholic acid (TLCA). Constipation group is defined as individuals with both functional constipation and IBS-C. Diarrhea group is defined as individuals with both functional diarrhea and IBS-D.

The subsequent ANOVA analysis showed that the fecal concentration of 10 of the 16 measurable bile acids, CA (*p* = 0.0002), CDCA (*p* = 0.001), GHDCA (*p* = 0.015), GDCA (*p* = 0.030), HCA (*p* = 0.025), GCA (*p* = 0.006), taurine (*p* = 0.018), TLCA (*p* = 0.007), TCDCA (*p* = 0.021), and TDCA (*p* = 0.018) were significantly different between healthy control, constipation (FC + IBS-C), and diarrhea (FD + IBS-D) groups (Figure 4). Post hoc analysis using the Wilcoxon test depicted as significance bars on boxplots showed that all ten bile acids were significantly higher in the diarrhea group compared to the constipation group. 

**Figure 5:**
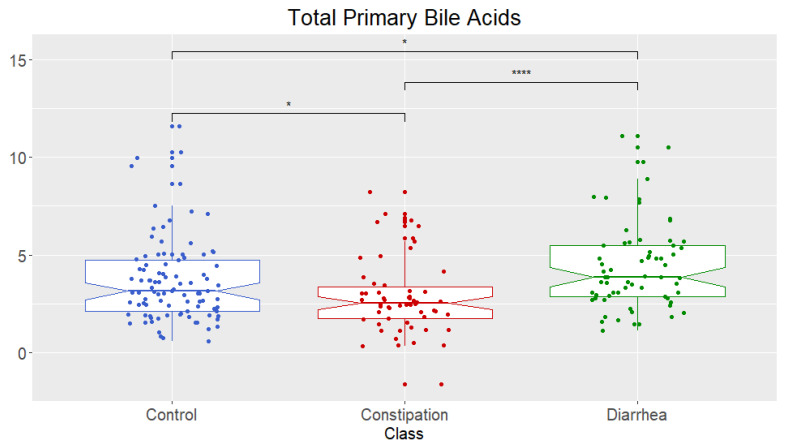
Total concentrations of fecal primary bile acids (sum of chenodeoxycholic acid (CDCA) and cholic acid (CA)) for healthy control and combined groups. Data presented as logged values of µg/mg of dried weight. Healthy control, constipation (FC + IBS-C) phenotype, diarrhea (FD + IBS-D) phenotype. Boxplots show median (center line), 25th and 75th percentile (top and bottom of boxes, respectively), with whiskers representing 1.5 times the inter-quartile range, and boundaries of notches show 95% confidential interval (CI). Statistical significance denoted as *p* < 0.05 (*), *p* < 0.001 (****). Constipation group is defined as individuals with both FC and IBS-C. Diarrhea group is defined as individuals with both FD and IBS-D.

Univariate analysis showed that the fecal concentration of total primary bile acids (sum of CA and CDCA) (Figure 5) was significant, and further pairwise mean comparisons showed there were significant differences between all three groups (healthy controls, constipation (FC + IBS-C), and diarrhea (FD + IBS-D)). Constipation (FC + IBS-C) was significantly lower than healthy controls and diarrhea (FD + IBS-D), and diarrhea (FD + IBS-D) significantly higher than healthy controls and constipation (FC + IBS-C).

**Figure 6:**
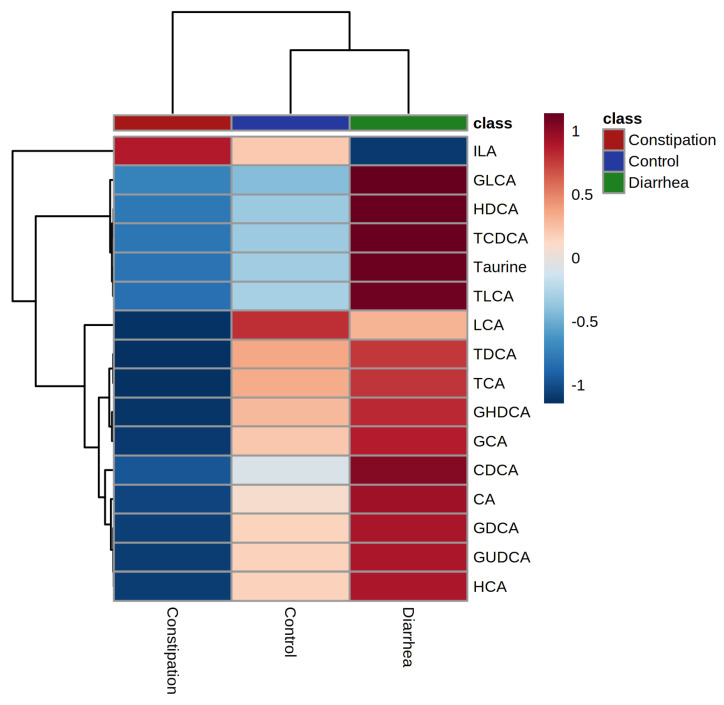
Hierarchical clustering analysis for average values of groups healthy control, constipation (FC + IBS-C) group, and diarrhea (FD + IBS-D) group. Data presented as z score of logged values of µg/mg. Color ribbon beneath upper dendrogram identifies group; healthy control—blue, constipation phenotype—red, diarrhea phenotype—green. Constipation group is defined as individuals with both functional constipation and IBS-C. Diarrhea group is defined as individuals with both functional diarrhea and IBS-D.

Hierarchical clustering analysis for average values of the fecal concentration of 16 bile acids (Figure 6) showed that the constipation (FC + IBS-C) group clustered separately from healthy controls and diarrhea (FD + IBS-D) groups which were clustered together. The heatmap highlighted a lower fecal concentration of all but one bile acid in the constipation (FC + IBS-C) group than in the diarrhea (FD + IBS-D) group. 

When investigating gender, two bile acids (GDCA *p* = 0.016, HDCA *p* = 0.003) were significantly higher in males compared to females. 

**Figure 7:**
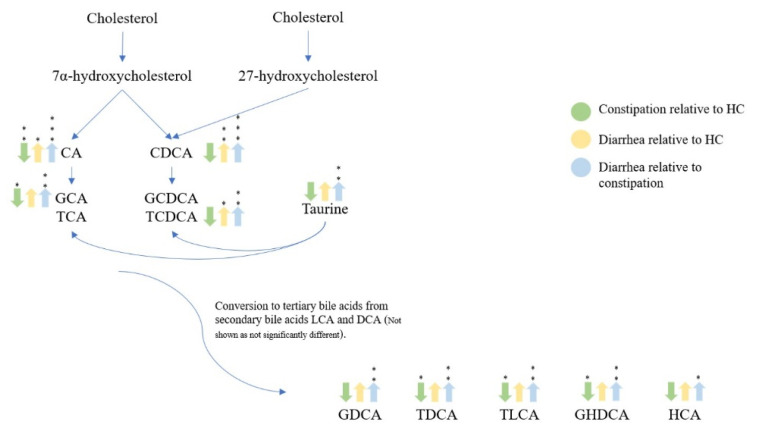
Bile acid pathway visualization showing significant increased or decreased concentrations in fecal samples for healthy control, constipation (FC + IBS-C), and diarrhea (FD + IBS-D) groups. Arrows depict whether the concentration is either up or down relative to the described color in the legend. Statistical significance denoted as *p* < 0.05 (*), *p* < 0.01 (**), *p* < 0.001 (***). Abbreviation: deoxycholic acid (DCA).

Pathway visualization of bile acid metabolites (Figure 7) summarized the significant differences (*p* < 0.05) between the constipation group (FC + IBS-C), diarrhea group (FD + IBS-D), and healthy control group. Reduced concentration of some fecal bile acid metabolites (CA, GCA, TCA, TCDA, TLCA, GHDCA) was observed in the constipation group (FC + IBS-C) compared to the control group. Similarly, the diarrhea group (FD + IBS-D) was characterized by an increased concentration in some bile acid metabolites (CA, CDCA, GCDCA, TCDCA) compared to the healthy control and the constipation group (FC + IBS-C) (CA, CDCA, GCA, TCA, GCDCA, TCDCA, GDCA, TDCA, TLDA, GHDCA, HCA). 

## 3. Discussion

This study reports the quantification of 16 bile acids in fecal samples from a cohort of participants across functional lower gut disorders. Quantitative analysis of 23 bile acids (including primary bile acids CA and CDCA) in fecal samples of the participants from this cohort was conducted and revealed that 16 bile acids were above detectable levels. The data showed that fecal concentrations of specific bile acids differed between individuals with functional gut disorders and healthy controls. Individuals with diarrhea (FD + IBS-D) were, in general, characterized by increased fecal excretion of bile acid metabolites (CA, CDCA, GCA, TCDCA, GDCA, TDCA, TLDA, GHDCA, HCA) compared to that of individuals with constipation (FC + IBS-C), IBS-M, and healthy controls. Individuals with functional diarrhea and constipation had similar bile acid concentration profiles to IBS-D and IBS-C, respectively.

The COMFORT cohort was predominantly female with similar age distributions between the phenotypes, reflective of worldwide rates of functional gut disorders. Analysis of fat and fiber intake, both of which could impact bile acid production, recorded as part of 3-day dietary diaries, showed no difference between the groups suggesting that differences in bile acid excretion were independent of diet and instead indicative of perturbed host or microbial mechanisms.

Fecal bile acids promote laxation [13,16]. The fecal concentration of CDCA and CA was higher in the combined diarrhea group (IBS-D + FD) compared to the combined constipation (IBS-C + FC) and healthy control groups, consistent with the findings of others [11,13]. The constipation group (IBS-C + FC) was characterized by a reduction in CA compared with healthy controls, unlike CDCA, where there was no difference in concentration. CDCA is produced from both primary and alternative pathways, while CA is produced solely via the primary pathway. This result suggests a possible dysfunction in the primary pathway in individuals with constipation (IBS-C + FC).

These findings suggest one of three mechanisms may be occurring. Either diarrhea (IBS-D + FD) and constipation (IBS-C + FC) individuals have perturbed biosynthesis or feedback regulating mechanisms, and therefore the known laxative effects of bile acids result in decreased colonic transit time and increased diarrhea. Alternatively, decreased colonic transit time could have reduced bile acid re-absorption from the luminal compartment into hepatic circulation, resulting in increased fecal bile acid concentrations in individuals with diarrhea as reported here. Others have suggested [10] that a cyclic process might occur where decreased re-absorption in the large intestine in participants with diarrhea initiates feedback mechanisms resulting in continuous production of bile acids. 

Previous studies support the finding that fecal and plasma bile acid concentrations differ within IBS subtypes [10,12,13]. However, they do not report values for concentration per mg/g of bile acids, but rather are focused on concentration differences compared to other groups. Our results show an increased concentration of fecal bile acids in those with IBS-D and FD, and a proportion of these individuals may have undiagnosed BAM [17], either as a cause or effect of diarrhea itself. The IBS-C and FC group was characterized by reduced fecal bile acids which could be linked to decreased fecal output and increased colonic transit, as previously described in other studies [8,11]. The findings suggest that FC and IBS-C or FD and IBS-D are functionally similar regarding bile acid metabolism.

Similarly to the findings reported here, previous studies have noted concentration differences in specific bile acids between healthy controls and IBS subtypes [8], although others have not [10]. Shin et al. [11] found no difference in total fecal bile acids, but reduced proportions of the primary bile acid CDCA and secondary bile acid deoxycholic acid in IBS-C and healthy control individuals. In contrast, Dior et al. [13] showed an increase in primary, but not secondary, fecal bile acids.

In the present study, the analysis of the 16 bile acids using hierarchical clustering and other supervised statistical tools (for example, partial least squares-discriminant analysis) could not reliably differentiate IBS participants within subtypes and from healthy participants according to their groupings based on the ROME IV criteria. Inherent variability and the difficulty with defining what makes a person ‘healthy’ could explain the lack of definitive clusters [18]. Classifying healthy participants based on responses to questionnaires means standardization can be difficult, ultimately highlighting the need for objectively measured scientifically validated biomarkers. Additionally, the functional basis of IBS exacerbates this, as even a healthy individual will experience gut ailments at certain times due to diet, stress, and other lifestyle factors. 

Primary bile acids (CA and CDCA), either measured separately or as a total combined concentration, could be accurately measured to distinguish between IBS subtypes. Although the concentration of other bile acids was altered, CA and CDCA were most different within the IBS subtypes. Additionally, when functional groups and IBS were combined into constipation or diarrhea groups, these same differences were observed, suggesting that the functional outcomes are similar between IBS and relevant functional groups.

The measurement of the primary bile acids, CA and CDCA, provides information at the start of the bile acid pathway where under-activation or over-activation of one pathway could increase or decrease shuttling through downstream bile acids. The relative concentrations of glycine and taurine conjugated compounds (GCA, TCA, GCDCA, and TCDCA) can provide a downstream view of the bile acid pathway. Measurement of glycine was not performed in this study. However, concentrations of taurine were different between healthy controls and IBS subtypes, perhaps highlighting differences in conjugation potential and suggesting that further analysis should include glycine. This analysis will make inferences about changes occurring downstream in the pathway and is likely important for a better understanding of, if and how these metabolites are involved in functional gut disorders. The combination of the analysis of primary bile acids in fecal samples with the analysis of predominantly ‘tertiary’ bile acids and microbial community changes would be necessary to advance the knowledge of the role of bile acids in functional gut disorders. 

The strengths of the analysis and data reported here are the quantitative LCMS method used to quantify the 23 bile acids, rather than total bile acids in stool samples from the COMFORT cohort representing the functional gut disorder spectrum. The sample size of the FD group was small in comparison to other groups. However, when combined with IBS-D participants, the group size was comparable to the other groups. The quantification of total bile acids in fecal samples is a proven method to diagnose BAM [17]. However, measuring total bile acids may provide limited insights into the physiological responses and mechanisms underlying functional gut disorders as the total will not equate to 100% of bile acids present [13]. Furthermore, considering the extensive microbial modification and epimerization results in a diverse range of bile acids and derived metabolites, obtaining standards to quantify all possible bile acids remains elusive. The data for total primary bile acids (CA and CDCA) reported here were accurately measured using internal standards.

There are also some limitations of this study relating to sample collection, dietary records, and sample analysis. Bile acids are metabolites that are influenced by dietary intake, host and microbial metabolism, and gut transit, and it was expected that some of these factors would impact the findings. Variations could arise as active recycling mechanisms will differ naturally between individuals. Additionally, the homogeneity of the samples could alter the concentration of bile acids. The home collection kit brings some potential sources of variation, such as differences in how long participants kept their sample out of the freezer or travel time on ice to the laboratory. The accuracy of the diet dataset relies on the participants accurately recording their dietary intake or when after food consumption bowel movements were performed.

## 4. Materials and Methods

### 4.1. Participants

Two hundred and fifty-nine individuals from Canterbury, New Zealand, were recruited to participate in The Christchurch IBS cohort to investigate mechanisms for gut relief and improved transit (COMFORT cohort. Universal trial number: U1111-1216-6662) cohort as previously described [19]. Cases were individuals with IBS, or a functional lower gut disorder diagnosis defined by the Rome Criteria IV (including Bristol stool score to identify subtypes; FC, FD, IBS-C, IBS-D, and IBS-M) undergoing colonoscopy for symptom investigation or surveillance aged 18–70 years. Healthy controls were asymptomatic individuals undergoing colonoscopy for surveillance due to a family history of colorectal cancer, personal history, or screening of colorectal cancer or polyps aged 18–70 years. Individuals that were pregnant or had a known organic disorder (inflammatory bowel disease, colorectal cancer, diverticulitis), previous bowel resection, and coeliac disease were excluded from the study. The study was approved by the University of Otago Human Ethics Committee (Ref. # H16/094). 

### 4.2. Diet Record and Sample Collection

Dietary records were kept for three sequential days (including one day of the weekend) before fecal collection [19]. Fecal samples were collected using at-home kits by participants, stored at 4 °C and transferred to the research facility within 24 h, where specimens were snap-frozen in liquid nitrogen and stored at −80 °C. Samples were transported to AgResearch, Palmerston North, New Zealand, on dry ice for bile acid analysis. Samples were freeze-dried and stored at −80 °C prior to extraction.

### 4.3. Standards and Reagents

Deuterated-cholic acid (d4-CA), bile acids (CA, CDCA, LCA, TCA, UDCA, taurine, βMCA, TαMCA, TβMCA, TLCA, TCDCA), and formic acid were purchased from Sigma-Aldrich Chemicals Co. (St Louis, MO, USA). All other bile acid standards (GCDCA, GCA, GDCA, GHDCA, GLCA, GUDCA, HCA, HDCA, ILA, TDCA, THDCA, TUDCA) were purchased from Steraloids Inc. (Newport, RI, USA). Acetonitrile (ACN) and methanol (MeOH) of Optima LC-MS grade quality were purchased from Thermo Fisher Scientific (Auckland, New Zealand).

### 4.4. Sample Extraction

Extraction methods followed those previously described by Joyce et al. [7] with minor modifications. Briefly, 100 mg of freeze-dried fecal samples were spiked with 100 ng of d4-CA and extracted with 700 µL ice-cold 50% MeOH in Eppendorf tubes pre-filled with 4 mm ceramic beads. The mixture was homogenized for six 30 s intervals (QIAGEN TissueLyser II, QIAGEN, Hilden, Germany) and incubated at −20 °C for 30 min and then centrifuged at 10,000× *g* for 25 min. Furthermore, 450 µL of the extract was transferred to a fresh tube and dried under nitrogen at 45 °C. One milliliter of ice-cold ACN containing 5% formic acid was added to each tube, and the sample briefly vortexed and agitated for 1 h gently at room temperature. The mixture was centrifuged at 10,000× *g* for 10 min and the resulting supernatant transferred to Eppendorf tubes and dried under nitrogen at 45 °C. The residual extract was dissolved in 150 µL of 50% MeOH, centrifuged at 10,000× *g* for 5 min and transferred to glass vials for chromatographic analysis. 

The analysis was completed on a SCIEX LCMS/MS QTRAP 6500+ system coupled to an ExionLC (SCIEX, Victoria, Australia). Furthermore, 1 µL of the sample was injected into a Waters Aquity Ultra Performance Liquid Chromatography (UPLC) column (Massachusetts, USA) maintained at 50 °C with a flow rate of 300 µL/min. The mobile phase, solvent A, consisted of 10 mM ammonium formate in H_2_O and solvent B, 10 mM ammonium formate, 5% ACN/95% MeOH. Gradient elution was as follows; 50% B held for 2 min then increased to 87% B at 13.5 min, 99% B at 18 min, returning to 50% B at 19 min and held until 21 min for re-equilibration. 

Mass spectral detection was performed in negative electrospray ionization mode using multiple reaction monitoring (MRM) for 23 bile acid compounds and the internal standard using electrospray ionization. Standards for all target compounds were run prior to sample analysis to optimize MRM conditions and separation of compounds. The source voltage was set to −4500 V, with a source temperature of 550 °C. Data was captured using Analyst (V1.6) software and processed on MultiQuant (V3.0.2) SCIEX software. Bile acid concentrations were generated from standard curves of standard injections for all 23 bile acids and the deuterated internal standard (d4-CA). Concentrations of bile acids were corrected to dry weight of fecal matter and are presented as µg/mg of dried fecal sample. K-nearest neighbor (KNN) was employed to input any missing values in the data using MetaboAnalyst (V4.0) [20,21]. 

### 4.5. Statistical Analyses

Residual plots and the Shapiro–Wilk test were employed to determine normality, showing uneven distribution, and thus the data were log-transformed. R statistical package (V3.6.1) was used for individual metabolite analyses and heatmap visualizations. ANOVA was used to compare means, with a probability (*p*) less than 0.05 deemed statistically significant. If a metabolite was significantly different, pairwise mean comparisons were used to compare differences between participant groups. Bile acid metabolite distributions were visualized using notched box plots, with the boundaries of the notches showing 95% confidence interval (CI). Metaboanalyst (V4.0) [20] was used for hierarchical clustering analysis (Ward’s Method clustering type). Basic nutritional data were analyzed using ANOVA to compare group differences in three-day dietary intake. Fecal dry weight was calculated relative to wet weight. 

## 5. Conclusions

In conclusion, this study shows that IBS subtypes combined with their respective functional groups have different fecal bile acid profiles compared to the healthy control group. Measuring fecal bile acid concentrations could not differentiate between functional groups and the respective IBS subtypes. Individuals with diarrhea (IBS-D + FD) showed increased fecal bile acid excretion compared to individuals with constipation (IBS-C + FC) and healthy controls, suggestive of a perturbed bile acid metabolism from that of a normal healthy gut. More specifically, concentration differences in primary bile acids in stool samples could be used to distinguish between constipation (IBS-C + FC) and healthy controls or between diarrhea (IBS-D + FD) and healthy controls. Host-microbial metabolism results in a diverse range of bile acids and derived metabolites. This study shows that bile acids have the potential to be utilized as biomarkers in the clinical setting. Although bile acid concentrations were not distinguishable between functional diarrhea and IBS-D, the study showed that diarrhea conditions are associated with increased bile acid excretion. Others showed that bile acid malabsorption could underlie many cases of diarrhea [10,11]. Therefore, understanding not just the total concentration of bile acids in the feces but also the relative concentrations may lead to more targeted use of established and novel bile acid sequestrants. Considering the microbial community and the physiological changes in the large intestine of these participants would help further advance the knowledge of the role of bile acids in functional gut disorders.

## Figures and Tables

**Table 1:** Bile acids analyzed using UPLC-MS with the corresponding acronym.

Name	Acronym
Beta-muricholic acid	βMCA
Cheno-deoxycholic acid	CDCA
Cholic acid	CA
Deuterated (d4) cholic acid (IS)	d4-CA
Glyco-cheno-deoxycholic acid	GCDCA
Glyco-cholic acid	GCA
Glyco-deoxycholic acid	GDCA
Glyco-hyo-deoxycholic acid	GHDCA
Glyco-litho cholic acid	GLCA
Glyco-urso-deoxycholic acid	GUDCA
Hyo-cholic acid	HCA
Hyo-deoxycholic acid	HDCA
Iso-lithocholic acid	ILA
Litho-cholic acid	LCA
Taurine	Taurine
Tauro-alpha-muricholic acid	TαMCA
Tauro-beta-muricholic acid	TβMCA
Tauro-cheno-deoxycholic acid	TCDCA
Tauro-cholic acid	TCA
Tauro-deoxycholic acid	TDCA
Tauro-hyo-deoxycholic acid	THDCA
Tauro-litho cholic acid	TLCA
Tauro-urso-deoxycholic acid	TUDCA
Urso-deoxycholic acid	UDCA

Bile acids with corresponding acronym.

**Table 2:** Characteristics of the participants of the COMFORT cohort used for the bile acid analyses, including average fecal dry weight percentage by subtypes.

	Control	IBS-C	FC	IBS-D	FD	IBS-M	*p*-Value
Female (male) *n*	52 (45)	23 (1)	25 (10)	40 (12)	11 (2)	24 (5)	0.0001
Age (mean)	54.4	53.5	59.1	52.8	58.4	50.5	0.128
Fecal average dry weight (%)	27.25	31.11	30.16	25.18	26.10	31.35	0.013

Abbreviations: Healthy control (control), functional constipation (FC), IBS-constipation (IBS-C), functional diarrhea (FD), IBS-diarrhea (IBS-D), IBS-mixed (IBS-M). *p* value for female (male) is significance between gender.

## Data Availability

Data will be made available on request.

## Appendix A

**Table A1:** Significance probability (*p*) values for bile acid metabolites between healthy control, constipation (FC + IBS-C) group and diarrhea (FD + IBS-D) group.

	Group *p*-Value	Gender *p*-Value	Group × Gender *p*-Value
CA	0.0002 ***	0.168	0.092
CDCA	0.001 ***	0.034	0.152
GHDCA	0.015 *	0.646	0.214
GUDCA	0.138	0.205	0.848
GDCA	0.030 *	0.286	0.016 *
HDCA	0.160	0.045	0.003 *
HCA	0.025 *	0.277	0.140
GCA	0.006 **	0.488	0.613
ILA	0.893	0.821	0.632
GLCA	0.295	0.765	0.250
Taurine	0.018 *	0.402	0.768
LCA	0.581	0.539	0.316
TLCA	0.007 **	0.348	0.653
TCDCA	0.021 *	0.320	0.456
TDCA	0.018 *	0.445	0.083
TCA	0.098	0.157	0.134

Statistical significance denoted as *p* < 0.05 (*), *p* < 0.01 (**), *p* < 0.001 (***). Abbreviations: cholic acid (CA); chenodeoxycholic acid (CDCA); glyco-hyo-deoxycholic acid (GHDCA); glyco-urso-deoxycholic acid (GUDCA); glyco-deoxycholic acid (GDCA); hyo-deoxycholic acid (HDCA); hyo-cholic acid (HCA); glyco-cholic acid (GCA); iso-lithocholic acid (ILA); glyco-litho cholic acid (GLCA); lithocholic acid (LCA); tauro-lithocholic acid (TLCA); tauro-cheno-deoxycholic acid (TCDCA); tauro-deoxycholic acid (TDCA); tauro-cholic acid (TCA). Constipation group is defined as individuals with both functional constipation and IBS-C. Diarrhea group is defined as individuals with both functional diarrhea and IBS-D.

**Table A2:** Average concentration values of groups for bile acid metabolites.

	Control	IBS-C	FC	IBS-D	FD	IBS-M
GHDCA	0.72 ± 3.61	0.29 ± 0.56	0.21 ± 0.26	0.34 ± 0.31	0.40 ± 0.49	0.24 ± 0.24
GUDCA	0.81 ± 3.88	0.32 ± 0.57	0.47 ± 1.26	0.40 ± 0.38	0.47 ± 0.58	0.28 ± 0.25
GDCA	110.41 ± 167.88	53.49 ± 54.29	126.42 ± 371.25	119.12 ± 130.64	91.47 ± 88.84	92.10 ± 112.37
HDCA	90.55 ± 101.77	92.99 ± 96.97	87.33 ± 77.63	122.15 ± 135.87	84.13 ± 88.98	71.12 ± 71.11
HCA	6.77 ± 13.63	2.26 ± 1.97	3.43 ± 3.87	5.62 ± 6.66	9.87 ± 21.42	5.21 ± 7.40
GCA	199.35 ± 317.56	68.1 ± 86.16	118.23 ± 222.78	221.83 ± 346.59	177.95 ± 169.83	166.30 ± 353.11
ILA	31.51 ± 20.67	30.91 ± 18.1	31.25 ± 17.92	33.17 ± 23.27	28.97 ± 15.79	35.01 ± 22.27
GLCA	0.18 ± 0.18	0.17 ± 0.13	0.21 ± 0.21	0.20 ± 0.15	2.01 ± 6.22	0.16 ± 0.11
Taurine	5.56 ± 18.91	4.65 ± 18.29	7.29 ± 23.0	17.85 ± 46.93	16.59 ± 29.4	1.84 ± 4.07
LCA	548.75 ± 336.88	483.52 ± 270.23	481.59 ± 271.46	618.36 ± 426.56	451.99 ± 189.5	513.83 ± 289.08
CDCA	29.65 ± 102.48	9.31 ± 15.35	11.61 ± 23.37	31.92 ± 67.42	32.39 ± 70.86	22.83 ± 50.26
CA	56.16 ± 255.46	12.44 ± 23.53	11.16 ± 30.87	58.79 ± 172.97	160.13 ± 513.21	37.52 ± 99.60
TLCA	0.94 ± 4.46	0.26 ± 0.77	0.35 ± 0.8	1.69 ± 6.10	2.17 ± 3.82	0.76 ± 3.06
TCDCA	3.35 ± 10.5	1.19 ± 2.48	4.65 ± 14.37	6.35 ± 19.67	7.28 ± 12.12	3.30 ± 8.97
TDCA	4.84 ± 12.5	0.64 ± 0.68	2.27 ± 6.24	3.54 ± 11.74	5.34 ± 5.89	3.26 ± 10.22
TCA	4.14 ± 7.82	2.06 ± 3.1	2.10 ± 4.87	6.47 ± 20.76	2.70 ± 4.0	6.55 ± 28.14

Values presented as mean (µg/mg) ± standard deviation. Abbreviations: Healthy control (control), functional constipation (FC), IBS-constipation (IBS-C), functional diarrhea (FD), IBS-diarrhea (IBS-D), IBS-mixed (IBS-M). Cholic acid (CA); chenodeoxycholic acid (CDCA); glyco-hyo-deoxycholic acid (GHDCA); glyco-urso-deoxycholic acid (GUDCA); glyco-deoxycholic acid (GDCA); hyo-deoxycholic acid (HDCA); hyo-cholic acid (HCA); glyco-cholic acid (GCA); iso-lithocholic acid (ILA); glyco-litho cholic acid (GLCA); lithocholic acid (LCA); tauro-lithocholic acid (TLCA); tauro-cheno-deoxycholic acid (TCDCA); tauro-deoxycholic acid (TDCA); tauro-cholic acid (TCA).
